# The Effects of Essential Oils on the Nervous System: A Scoping Review

**DOI:** 10.3390/molecules28093771

**Published:** 2023-04-27

**Authors:** Apsorn Sattayakhom, Sineewanlaya Wichit, Phanit Koomhin

**Affiliations:** 1 School of Allied Health Sciences, Walailak University, Nakhonsithammarat 80160, Thailand; apsorn.sa@wu.ac.th; 2 Center of Excellence in Innovation of Essential Oil and Bioactive Compounds, Walailak University, Nakhonsithammarat 80160, Thailand; 3 Department of Clinical Microbiology and Applied Technology, Faculty of Medical Technology, Mahidol University, Nakhon Pathom 73170, Thailand; sineewanlaya.wic@mahidol.ac.th; 4 School of Medicine, Walailak University, Nakhonsithammarat 80160, Thailand

**Keywords:** essential oil, the nervous system, aromatherapy

## Abstract

Essential oils are a mixture of natural aromatic volatile oils extracted from plants. The use of essential oils is ancient, and has prevailed in different cultures around the world, such as those of the Egyptians, Greeks, Persians, and Chinese. Today, essential oils are used in traditional and complimentary medicines, aromatherapy, massage therapies, cosmetics, perfumes and food industries. The screening effect of essential oils has been studied worldwide. They demonstrate a range of biological activities, such as antiparasitic, antifungal, antibacterial, antiviral, antioxidant, anti-inflammatory, anticancer, antiaging, and neuroprotective properties. In this scoping review, we provide a 10-year updated comprehensive assessment of volatile oils and their effects on the nervous system. MEDLINE, Scopus, and Google Scholar were systematically and strategically searched for original studies investigating these effects from 2012 to 2022. Approximately seventy studies were selected as included studies. Among these studies, several outcomes were reported, including antistress, antianxiety, analgesic, cognitive, and autonomic effects. Some essential oils showed developmental benefits, with the potential to induce neurite outgrowth. The neurotransmitter receptor level can also be modified by essential oil application. Physiological and pathophysiological outcome measures were reported. For physiological outcomes, arousal, cognitive performance, circadian eating behavior, emotional modulation, consumer acceptance, preferences, and willingness to buy were investigated. For pathophysiological conditions, pain, depression, anxiety, stress, sleep disorder, mental fatigue, agitated behavior, and quality of life were measured. In conclusion, essential oils showed promising effects on the nervous system, which can be further applied to their use in functional foods, drinks, and alternative therapy.

## 1. Introduction

Essential oils (EOs) are the naturally aromatic oily liquids produced by plants, which are responsible for their essence or odor. EOs are found in great quantities in plant oil sacs or oil glands. EOs can be extracted from different parts of plants, including their leaves, barks, flowers, buds, seeds, and peels. As EOs are a concentrated hydrophobic mixture of hydrocarbon volatile compounds that easily evaporate at room temperatures, they are also known as volatile oils. Thus, inhalation via the olfactory system is a common method of use. During the extraction, most volatiles oil are commonly and easily isolated through steam- or hydro-distillation methods. However, for the oils produced from the rind of fruits such as those in the citrus family, the cold-pressed or expressed oils extraction method is usually used. A solvent extraction method is also used for some plant materials that cannot tolerate heat (in steam) or be subjected to cold-pressing, such as the rose, neroli, tuberose, jasmine, and oak [1]. In most plants, the major components of EOs are terpenoid and phenylpropanoid derivatives. Terpenoids are the main components (comprising approximately 80%), but phenylpropanoid provides the flavor and odor of the EOs. Terpenoid and phenylpropanoid are derived from different primary metabolic precursors by different biosynthetic pathways. The pathways for terpenoids synthesis are mevalonate and mevalonate independent (deoxyxylulose phosphate), whereas phenylpropanoids synthesis occurs through the shikimate pathway [2,3]. The scent and bioactivity of EOs depends on their chemical compositions. Different EOs from different plant species and habitats have various potentials. According to the various biological properties, EOs have been widely used and have attracted increased attention in recent years. The primary use of EOs started in the medical field. The term “essential oil” comes from “quinta essentia” in ancient Latin, which means the fifth element. The fifth element is the spirit or life force integrated with the other four elements: fire, air, earth, and water. The isolation of essential oil was thought to be the process of removing the spirit from the plant and the oils were used as healing essences with medical benefits [3]. Until now, the use of EOs has been studied and demonstrated across a range of biological activities, exhibiting antiseptic, antibiotic, antifungal, antiviral, anti-inflammatory, antioxidants, anti-cancer, antinociceptive, carminative, laxative, rubefacient, antidepressant, anticonvulsant, analgesic, sedative, and immunomodulatory properties [4]. EOs have been widely applied in plants and animals industries, perfumery, cosmetics, food, and pharmaceuticals. The popularity of EOs and aromatic plants is also continuously growing according to their various activities and the increase in consumer demand. The use of EOs for the human body or routes of administration occurs not only via inhalation, but also including skin absorption (topical or aromatherapy), as well as ingestion. However, inhalation via the olfactory system is the fastest and easiest method. It has been reported that EOs affect the immediate changes in the autonomic nervous system and physiological responses such as pupil dilation, blood pressure, muscle tone, pulse rate, skin temperature, and brain activity. These body responses improve physical, mental, and emotional well-being after 15 min of inhalation [5,6,7]. The component of EOs is detected by the olfactory receptors on a nasal olfactory epithelium, which causes the stimulation of olfactory nerves and transmission of a signal to the central nervous system, including the limbic system and hypothalamus, which further modulate human behavior and body function [7]. These indicate that the nervous system is the very first mechanism of the body’s response to EOs. There has been a lot of research in this growing field of study. However, there is no updated summary or review describing the physiological and pathophysiological outcomes of the nervous system. There are a limited number of systematic review articles in the database providing more specific topics such as the pharmacological properties of EOs or confined health outcomes. The systematic scoping review will show more broad outcomes from basic physiology to more sophisticated pathophysiology in animal and human research. Therefore, we updated and discussed the 10-year evidence related to EOs in relation to the physiological and pathophysiological outcomes of the nervous system in a systematic scoping review.

## 2. Results

### 2.1. Essential Oils and Application Methods

**Figure 1:**
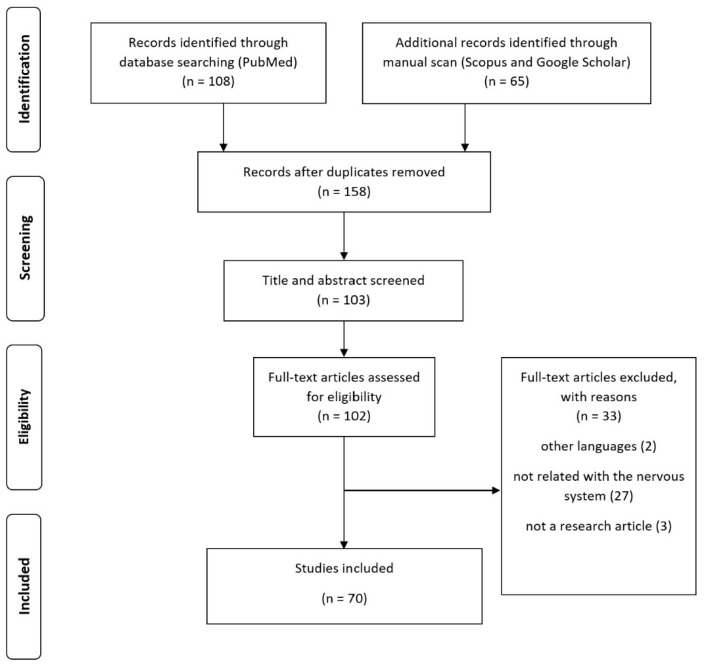
Flow chart of the study identification and selection process.

According to the included studies, the effects of essential oils on the nervous system have been widely studied in every region around the world. Several types of essential oils were used in the included studies. In this scoping review, we included both animal and human studies to comprehensively understand the possible mechanisms to outcome measures in humans. The PubMed database showed more specific results than others; however, Scopus and Google Scholar showed more sensitivity than PubMed (Figure 1). Based on the PubMed database, 81.43% of the included studies were conducted on humans, 18.57% were carried out on animals and about 1.43% were researched on cell cultures. Lavender essential oil was the most used essential oil, appearing in approximately 30.71% of the studies. The second most popular category of essential oil in the studies is the *Citrus* spp., which includes essential oils such as orange, bergamot, grapefruit, and lemon (24.4%). Rosemary essential oil was the third choice after the lavender oil and *Citrus* spp. oil (5.51%). Mint, rose, cedar wood, geranium, lemon grass, chamomile, cinnamon, and others were also used, though in smaller amounts. The routes of administration used in the included studies including inhaled, oral, topical, massage, injection, and immersion. Inhalation was the most popular route of administration at approximately 58.57%. There was a wide range of study populations and age groups, as reported in Table 1.

### 2.2. Physiological Outcomes

Several levels of physiological hierarchy were investigated (Table 2). At the very first level of alertness, electroencephalography was used to evaluate the arousal and sedative properties in animals and humans. Lavender oil showed a sedative effect. Peppermint and coffee showed a stimulating effect, as shown by the electroencephalogram. The autonomic nervous system was frequently measured after the alertness level. Heart rate (HR), blood pressure (BP), respiratory rate (RR), and heart rate variability (HRV) were evaluated. Lavender, rosemary, bergamot, eucalyptus, rose, yuzu, lemon, Meniki, Hinoki, *Juniperus phoenicea* gum extract, *Copaifera officinalis* (Balsam Copaiba) resin, *Aniba rosaeodora* (Rosewood) wood oil, *Juniperus virginiana* oil, grapefruit oil, and petitgrain revealed autonomic nervous system (ANS) activity modification via the inhalation route. A massage with lavender and geranium oils also affected the ANS by reducing heart rate and blood pressure after the massage. In the opposite of alertness, lavender improved objective sleep quality. For another type of alertness and arousal, peppermint, rosemary, grapefruit, and cinnamon oils improved vigilance using a vigilance test. In response to olfactory stimuli, the cortisol level and salivary chromogranin A were affected by lavender, bergamot, yuzu, *Juniperus phoenicea* gum extract, *Copaifera officinalis* resin, *Aniba rosaeodora* wood oil, *Juniperus virginiana* oil, and grapefruit essential oil.

In addition to the objective measure of stress hormones, these oils also affected subjective emotional measurements. Interestingly, most essential oil use decreased stress and negative emotions with the reduction in stress hormones and parasympathetic stimulation. Anxiolytic effects were reported in lavender, *Juniperus phoenicea* gum extract, *Copaifera officinalis* resin, *Aniba rosaeodora* wood oil, *Juniperus virginiana* oil, *Origanum majorana*, *Citrus sinensis*, and petitgrain. At the uppermost levels, cognitive functions and behaviors were finally affected by essential oils. Rosemary essential oil improved cognition using a computerized cognitive task. Spearmint and peppermint essential oils modulated performance during a demanding cognitive task and reduced mental fatigue during a prolonged cognitive task. Petitgrain essential oil could improve performance in the workplace when added to an aroma diffuser in the work room. Oregano and rosemary essential oils increased consumer acceptability and willingness to buy food products. Among the positive results, there were also negative results. Lavender could not modulate stress and ANS responses in patients with coronary bypass surgery, which only affected systolic blood pressure. Koteless and Babulka also showed negative effects of rosemary, lavender, and eucalyptus oils on adult volunteers.

### 2.3. Pathophysiological Outcomes

Depression was the most studied clinical manifestation related to the effect of essential oils. Lavender, chamomile, bergamot, sweet orange, anise, geranium, and mountain pepper reduced depression in the elderly, postpartum women, restless patients, breast cancer patients, irritable bowel syndrome patients, mixed anxiety and depressive disorder, and residents in a long-term care unit. Analgesic and anxiolytic effects were the second most attractive topics. Lavender, bergamot, and *Melissa officinalis* (lemon balm) showed an analgesic effect, which can be observed in mice, rats, neonatal, premature babies, and women with dysmenorrhea. Lavender, bergamot, geranium, mountain pepper, chamomile, *Juniperus phoenicea* gum extract, *Copaifera officinalis* resin, *Aniba rosaeodora* wood oil, *Juniperus virginiana* oil, and *Citrus* spp. oil showed anxiolytic activity in several types of populations. Other interesting outcomes in different groups of patients were reported as described in Table 3. Essential oils could alleviate fatigue, memory problems, behavioral symptoms, stress, inhalant cravings, and sleep problems without the potential for abuse. They still showed negative effects on several groups of breast cancer patients undergoing breast reconstruction, children with burns, mild to moderate dementia sufferers older than 65 years old, and coronary bypass surgery patients.

**Table 1:** Characteristics of included studies.

Author	Year	Country	Study Design	Study Population	Age (Mean ± SD or Range)	Sample Size (n)
Scuteri et al. [8]	2022	Italy	experimental study	male ddY mice	2 months	6
Pereira et al. [9]	2022	Portugal	randomized control trial (RCT)	patients diagnosed with Breast cancer, stage I and II	51.48 ± 10.34 years	56
Maya-Enero [10]	2022	Spain	RCT	neonatals	3–6 months	71
Hawkins et al. [11]	2022	United States (US)	RCT	post-COVID-19 female participants	19–49 years	19
Ebrahimi et al. [12]	2022	Iran	RCT	elderly participants	72.81 ± 7.14 years	61
Du et al. [13]	2022	Canada	RCT	healthy university students	22.80 years	59
Dehghan et al. [14]	2022	Iran	RCT	patients undergoing chronic hemodialysis	53.66 ± 12.30 years	86
Chen et al. [15]	2022	Taiwan	RCT	postpartum women	>20 years	29
Atef et al. [16]	2022	Egypt	experimental study	male Wistar rats	8 weeks	50
Usta et al. [17]	2021	Türkiye	RCT	premature babies	24–37 weeks	31
Shammas et al. [18]	2021	US	RCT	patients diagnosed with breast cancer and undergoing microvascular breast reconstruction.	32–68 years	27
Sgoifo et al. [19]	2021	Italy	RCT	healthy women participants	32.70 ± 1.80 years	20
Seifritz et al. [20]	2021	Canada	RCT	healthy male or female participants	18–55 years	34
Schneider [21]	2021	Germany	RCT	women and men participants	34.20 ± 6.90 years	15
Mascherona et al. [22]	2021	Switzerland	RCT	patients diagnosed with dementia and behavioral and psychological symptoms of dementia (BPSD)	87.06 ± 6.95 years	16
Manor et al. [23]	2021	Thailand	experimental study	adult male Wistar rats	2 months	not available (n/a)
Ko et al. [24]	2021	Taiwan	experimental study	healthy male or female participants	22 ± 2 years	9
Karimzadeh et al. [25]	2021	Iran	RCT	conscious patients admitted to ICUs	36.41 ± 12.06 years	56
Ferreira et al. [26]	2021	Brazil	experimental study	juveniles *Oreochromis niloticus*	6 weeks	12
Takahashi et.al. [27]	2020	Japan	RCT	Alzheimer type dementia patients	76.20 ± 9.80 years	19
Schneider [28]	2020	Germany	RCT	male or female participants	24–52 years	7
Hacke et al. [29]	2020	Brazil	experimental study	adult zebrafish	4–6 months	14
Kawai et al. [30]	2020	Japan	experimental study	healthy men participants	21 ± 2.10 years	13
Bae et al. [31]	2020	US	RCT	recruited residents in long term care unit	81.24 ± 11.05 years	29
Watson et al. [32]	2019	Australia	RCT	nursing home residents diagnosed with dementia	89.31 ± 6.30 years	49
Son et al. [33]	2019	Korea	RCT	sophomore female nursing students	20 years	32
Patra et al. [34]	2019	Germany	experimental study	female Suffolk sheep	121 ± 3.70 days	12
Park et al. [35]	2019	Korea	quasi-experimental study	healthy female Korean participants	21–39 years	12
Felipe et al. [36]	2019	Brazil	experimental study	male Swiss albino mice	2 months	6
Xiong et al. [37]	2018	China	RCT	community-dwelling adults with symptoms of depression	67.87 ± 7.51 years	20
Vital et al. [38]	2018	Brazil	experimental study	students, employers, and visitors	group of age 18–24, 25–39, 40–54, and >55 years	10
Van Dijk et al. [39]	2018	South Africa	RCT	children admitted to the burns unit	0–13 years	110
Senturk and Kartin [40]	2018	Türkiye	RCT	hemodialysis patients	≥30 years	17
Qadeer et al. [41]	2018	Pakistan	experimental study	locally breed albino Wistar rats	2 months	6
Moss et al. [42]	2018	United Kingdom (UK)	RCT	healthy female and male adults	22.84 ± 3.95 years	40
Montibeler et al. [43]	2018	Brazil	RCT	female nursing team of a surgical center	39.50 ± 9.87 years	19
Kennedy et al. [44]	2018	UK	RCT	male or female participants	21–35 years	24
Kalayasiri et al. [45]	2018	Thailand	RCT	male participants with inhalant dependence	27.9 ± 5.77 years	17
Brnawi et al. [46]	2018	US	experimental study	male or female participants	36 ± 14 or 21–65 years	75
Mosaffa-Jahromi et al. [47]	2017	Iran	RCT	participants with irritable bowel syndrome with mild to moderate depression	34.15 ± 9.29 years	40
Matsumoto et al. [48]	2017	Japan	RCT	women with subjective premenstrual symptoms	20.60 ± 0.20 years	10
Lam et al. [49]	2017	Hongkong	in vitro study	pheochromocytoma PC12 cells	-	3
Karadag et al. [50]	2017	Türkiye	RCT	male and female patients in coronary ICU	50.33 ± 12.14 years	30
Huang and Capdevila [6]	2017	Spain	RCT	administrative university workers	42.21 ± 7.12 years	42
Goepfert et al. [51]	2017	Germany	RCT	conscious and non-conscious palliative patients	42−84 years	20
Forte et al. [52]	2017	Italy	experimental study	pigs	35–220 days	72
Chen et al. [53]	2017	Taiwan	RCT	healthy pregnant women	33.31 ± 4.01 or 24–43 years	24
Kasper et al. [54]	2016	Germany	RCT	male and female outpatients diagnosed with mixed anxiety and depressive disorder	18–65 years	160
Gaston et al. [55]	2016	Argentina	experimental study	male and female meat-type chicks	1 day old	16
Dyer et al. [56]	2016	UK	experimental study	patients diagnosed with sleep problems	16–84 years	65
Yoshiyama et al. [57]	2015	Japan	RCT	patients with mild to moderate dementia in a nursing home	≥65 years	7
Watanabe et al. [58]	2015	Japan	RCT	healthy females	21.3 ± 1.02 or 20–23 years	7
Kasper et al. [59]	2015	Germany	RCT	male and female out-patients with a diagnosis of restlessness	18–65 years	86
Hasanein and Riahi [60]	2015	Iran	experimental study	locally bred male Wistar rats	2 months	8
Chen et al. [61]	2015	Taiwan	quasi-experimental study	healthy adults	20–21 years	16
Bikmoradi et al. [62]	2015	Iran	RCT	patients undergone coronary artery bypass graft	65.13 ± 9.76 years	30
Nagata et al. [63]	2014	Japan	RCT	male and female asymptomatic participants undergoing screening computed tomography colonography	45–59 years	56
Matsumoto et al. [64]	2014	Japan	RCT	healthy women participants	20.50 ± 0.10 years	20
Koteles and Babulka [65]	2014	Hungary	quasi-experimental study	male adult participants	37.70 ± 10.90 years	33
Kasper et al. [66]	2014	Germany	RCT	male and female with generalized anxiety disorder	18–65 years	128
Igarashi et al. [67]	2014	Japan	quasi-experimental study	female university and graduate students	21.60 ± 1.50 or 19–26 years	19
Baldinger et al. [68]	2014	Austria	RCT	healthy participants	25.60 ± 3.70 years	17
Varney and Buckle [69]	2013	US	RCT	male and female participants	25–45 years	7
Taavoni et al. [70]	2013	Iran	RCT	postmenopausal participants	45–62 years	30
Seol et al. [71]	2013	Korea	RCT	female patients diagnosed with urinary incontinence	33–75 years	12
Igarashi [72]	2013	Japan	RCT	28-week-pregnant women	29.30 ± 4.30 years	6
Han et al. [73]	2013	China	experimental study	male ICR mice	2 months	10
Fu et al. [74]	2013	Australia	RCT	patients diagnosed with dementia	84 ± 6.36 years	22
Brito et al. [75]	2013	Brazil	experimental study	male adult albino Swiss mice	3 months	6
Apay et al. [76]	2012	Türkiye	quasi-experimental study	midwifery and nursing students	20.31 ± 1.09 years	44

**Table 2:** Effects of essential oils on physiological responses.

Author	Essential Oils	Application Methods	Measures	Outcomes
Du et al. [13]	lemon and grapeseed	inhaled	cognitive function tests	shortened reaction time response, more impulsive decision-making
Dehghan et al. [14]	lavender, rosemary, and orange	inhaled	retrospective and prospective memory scale	only lavender or rosemary can reduce some memory problems in hemodialysis patients by reduction of retrospective memory problems
Sgoifo et al. [19]	*Juniperus phoenicea* gum extract, *Copaifera officinalis* (Balsm Copaiba) resin, *Aniba rosaeodora* (Rosewood) wood oil and *Juniperus virginiana* oil	dermal	psychological questionnaires (anxiety, perceived stress, and mood profile), autonomic parameters (heart rate (HR) and heart rate variability (HRV)), and neuroendocrine (salivary cortisol) measurements	stress resilience due to favorable physiological, neuroendocrine, and psychological effects
Schneider [21]	peppermint, rosemary, grapefruit, and cinnamon	inhaled	vigilance test using computerized attention and concentration tests	improved vigilance
Manor et al. [23]	lavender	inhaled	electroencephalogram (EEG)	distinct anxiolytic-like effects and sleep enhancing purpose
Ko et al. [24]	lavender	inhaled	sleep laboratory: EEG, electromyogram (EMG) and electrooculogram (EOG) signals	improved subjective and objective sleep qualities
Kawai et al. [30]	grapefruit	inhaled	muscle sympathetic nerve activity (MSNA), blood pressure (BP), heart rate (HR), and cortisol concentration	changed in BP, muscle sympathetic nerve activity changed, decreased stress hormone (cortisol) concentration
Park et al. [35]	lavender, peppermint, and coffee	inhaled	quantitative and objective EEG and the questionnaire	stabilized for lavender and aroused for peppermint and coffee
Vital et al. [38]	oregano and rosemary	inhaled/oral	a 9-point scale	higher consumer acceptance and willingness to buy
Moss et al. [42]	rosemary	oral	computerized cognitive tasks	enhance cognition
Montibeler et al. [43]	lavender and geranium	massage	biophysiological and psychological parameters	reduction in heart rate and blood pressure levels after massage sessions
Kennedy et al. [44]	spearmint and peppermint	oral	neurotransmitter receptor binding, acetylcholinesterase (AChE) inhibition, mood scales, and standardized cognitively demanding tasks	peppermint with high levels of menthol characteristic as in vitro cholinergic inhibitory, calcium regulatory, GABA/nicotinic binding/modulated performance on demanding cognitive task/attenuated the increase in mental fatigue associated with extended cognitive task
Brnawi et al. [46]	cinnamon bark and leaf	oral	a 9-point hedonic scale	natural antimicrobial ingredient in milk beverages—sensory aspect
Matsumoto et al. [48]	yuzu and lavender	inhaled	heart rate variability and the profile of mood states (POMS) questionnaire	alleviated premenstrual emotional symptoms and improved parasympathetic nervous system activity
Huang and Capdevila [6]	petitgrain	inhaled	the stait–trait anxiety inventory (STAI) questionnaire, POMS questionnaire, and HRV	improved performance in the workplace-autonomic balance, reduced stress level, and increased arousal level-attentiveness-alertness
Goepfert et al. [51]	lemon and lavender	inhaled	physiological parameters: respiratory rate (RR), heart rate (HR), systolic (SBP) and diastolic pressure (DBP)	lemon increased RR, HR, DBP, and lavender decreased RR
Forte et al. [52]	oregano	oral	sensory analysis of the consumer tests	improved consumer perception of the meat quality
Chen et al. [53]	lavender	massage	salivary cortisol and immune function measurements	decreased stress and enhanced immune function
Watanabe et al. [58]	bergamot	inhaled	salivary cortisol level	lower salivary cortisol compared to rest
Chen et al. [61]	Meniki and Hinoki wood	inhaled	subject’s BP, HR, HRV, sympathetic and parasympathetic nervous system (SNS and PSNS), and POMS questionnaire	simulated a pleasant mood status-regulators of sympathetic nervous system dysfunctions
Bikmoradi et al. [62]	lavender	inhaled	DASS-21 questionnaire, HR, RR, SBP and DBP	no effects on mental stress and vital signs in patients following coronary bypass surgery (CABG), but has possibly significant effect on systolic blood pressure of patients
Nagata et al. [63]	bergamot	inhaled	a visual analog scale	showed little effect on pain, discomfort, vital signs, as well as preferred music and aroma during the next computed tomography (CT)
Matsumoto et al. [64]	yuzu	inhaled	POMS questionnaire and salivary chromogranin A	alleviated negative emotional stress-suppression of sympathetic nervous system activity
Koteles and Babulka [65]	rosemary, lavender, and eucalyptus	inhaled	EEG, HR, BP, HRV and self-reported questions and statements alertness, pleasantness, expectations, and perceived effect	no effect on any assessed variables (HR, BP, and HRV) and perceived subjective changes-non-conscious states
Igarashi et al. [67]	rose	inhaled	HRV and subjective evaluations	induced physiological–psychological relaxation
Baldinger et al. [68]	lavender	oral	positron emission tomography (PET) and magnetic resonance imaging (MRI) measurements	the anxiolytic effects of Silexan via serotonin-1A receptor
Igarashi [72]	lavender, petitgrain, and bergamot	inhaled	POMS questionnaire and autonomic nervous system parameters	no major differences observed between the two groups but essential oils containing linalyl acetate and linalool effective for the POMS and parasympathetic nerve activity based on an intragroup comparison
Brito et al. [75]	citronellol	paw injection	nociceptive test	attenuated orofacial pain
Son et al. [33]	sweet marjoram and sweet orange	inhaled	participants’ Foley catheterization skill, the Korean version of the revised test anxiety scale, and a numeric rating score	improved the performance of fundamental nursing skills and reduced anxiety and stress

**Table 3:** Effects of essential oils on pathophysiological responses.

Author	Essential Oils	Application Methods	Measures	Outcomes
Scuteri et al. [8]	bergamot	inhaled	licking/biting behavior	analgesic properties
Pereira et al. [9]	bergamot, geranium, and mountain pepper	inhaled	the relationship between anxiety, depression, and quality of life (primary outcomes), as well as the impact of hedonic aroma	long-term emotional and quality of life-related adjustment
Maya-Enero [10]	lavender	inhaled	pain assessment	decreased crying time
Hawkins et al. [11]	thyme, orange peel, clove bud, and frankincense	inhaled	multidimensional fatigue symptom inventory	lowered fatigue score
Ebrahimi et al. [12]	lavender and chamomile	inhaled	the depression, anxiety, and DASS stress-scale	both lavender and chamomile essential oils helped decrease depression, anxiety, and stress levels
Chen et al. [15]	bergamot	inhaled	questionnaire including the Edinburgh postnatal depression scale and postpartum sleep quality scale (PSQS)	alleviated depressive mood in postpartum
Usta et al. [17]	lavender	inhaled	pain scores	pain control in premature infants during heel lancing
Shammas et al. [18]	lavender	inhaled	hospital anxiety and depression scale, Richards–Campbell sleep questionnaire, and the visual analogue scale for quantifying stress, anxiety, depression, sleep, and pain	no measurable advantages in breast reconstruction
Sgoifo et al. [19]	*Juniperus phoenicea* gum extract, *Copaifera officinalis* (Balsm Copaiba) resin, *Aniba rosaeodora* (Rosewood) wood oil and *Juniperus virginiana* oil	topical	psychological questionnaires (anxiety, perceived stress, and mood profile), autonomic parameters (heart rate (HR) and heart rate variability (HRV)), and neuroendocrine (salivary cortisol) measurements	stress resilience due to favorable physiological, neuroendocrine and psychological effects
Seifritz et al. [20]	lavender	oral	a short form of the addiction research center inventory (visual analogue scales assessing positive, negative, and sedative drug effects)	no abuse potential
Mascherona et al. [22]	lavender and sweet orange	inhaled	measures the stress felt by professional caregiver using Italian version of the NPI-NH scale	might improve wellbeing of patients and caregivers
Karimzadeh et al. [25]	lavender and citrus	inhaled	the state subscale of State-Trait Anxiety Inventory	reduced the anxiety of patients admitted to ICUs
Ferreira et al. [26]	*Ocimum gratissimum*	water medication /immersion	the time of anesthesia induction and recovery during anesthesia of *Oreochromis niloticus* exposed to essential oil of *Ocimum gratissimum*	reduced the stress of transport, and improved the oxidative status of *Oreochromis niloticus* by stable plasma glucose and change antioxidant defense system by increasing hepatic and kidney ROS
Takahashi et.al. [27]	cedar	inhaled	the neuropsychiatric inventory (NPI), the Japanese version of Zarit Caregiver Burden interview (J-ZBI), and the Alzheimer’s Disease Assessment Scale-cognitive subscale (ADAS-cog).	improved behavioral and psychological symptoms of dementia
Hacke et al. [29]	lemongrass, pure citral and geraniol	water medication/immersion	the light–dark test	anxiolytic effect
Bae et al. [31]	lavender	inhaled	the geriatric depression scale (GDS)	positive distraction during the healing process-theory of supportive design
Watson et al. [32]	lavender and lemon balm	inhaled	NPI and Cohen-Mansfield agitation inventory (CMAI)	reduced agitated behavior in residents without dementia, but no reduction with treatments when compared to placebo independent of cognitive groups
Xiong et al. [37]	lavender, sweet orange, and bergamot	massage	the geriatric depression scale (GDS)	intervened depression in older adults
Van Dijk et al. [39]	chamomile, lavender, and neroli	massage	the behavioral relaxation scale and the COMFORT behavior scale	not effective in reducing stress of children with burns
Senturk and Kartin [40]	lavender	inhaled	Pittsburgh sleep quality index, the Hamilton anxiety assessment scale, and visual analog scale for daytime sleepiness level	improved sleep problems and anxiety for dialysis nurses
Kalayasiri et al. [45]	lavender and synthetic oil	inhaled	the modified version of Penn alcohol craving score for inhalants	reduced inhalant craving
Mosaffa-Jahromi et al. [47]	anise	oral	the Beck Depression Inventory Scale II	reduction of total score of Beck Depression Inventory II in depressed patients with irritable bowel syndrome
Matsumoto et al. [48]	yuzu and lavender	inhaled	HRV and the profile of mood states (POMS) questionnaire	alleviated premenstrual emotional symptoms and improved parasympathetic nervous system activity
Karadag et al. [50]	lavender	inhaled	Pittsburgh sleep quality index and the Beck anxiety inventory scale.	increased quality of sleep and reduced level of anxiety in coronary artery disease patient
Kasper et al. [54]	lavender	oral	Hamilton anxiety rating scale and the Montgomery Asberg depression rating scale	improved impaired daily living skills and health-related quality of life
Dyer et al. [56]	bergamot, sandalwood, frankincense, mandarin, lavender, orange sweet, petitgrain, lavandin, mandarin, bergamot, lavender, and roman chamomile.	inhaled	a patient questionnaire	improved Likert scale measuring sleep quality
Yoshiyama et al. [57]	bitter orange leaf, *Cymbopogon martini*, *Picea mariana*, lavender, damask rose, grapefruit, and lemon balm	massage	behavioral and psychological symptoms of dementia (BPSD) and activities of daily living (ADLs)	no improvement of BPSD-ADLs with dementia
Kasper et al. [59]	lavender	oral	the Hamilton anxiety rating scale, Pittsburgh sleep quality index, and the Zung Self-rating anxiety scale	calming and anxiolytic efficacy
Hasanein and Riahi [60]	lemon balm	injection	nociceptive test	treatment of painful diabetic neuropathy
Bikmoradi et al. [62]	lavender	inhaled	DASS-21 questionnaire, HR, RR, systolic (SBP) and diastolic pressure (DBP)	no effects on mental stress and vital signs in patients following coronary bypass surgery (CABG), but has a possibly significant effect on systolic blood pressure in patients
Nagata et al. [63]	bergamot	inhaled	a visual analog scale	showed little effect on pain, discomfort, vital signs, as well as preferred music and aroma during the next CT
Kasper et al. [66]	lavender	oral	Hamilton anxiety scale, Covi anxiety scale, Hamilton rating scale for depression, and clinical and global impressions	antidepressant effect/improved general mental health–health-related quality of life
Baldinger et al. [68]	lavender	oral	PET and MRI measurements	the anxiolytic effects of Silexan via serotonin-1A receptor
Varney and Buckle [69]	jojoba oil, peppermint, basil, and helichrysum	inhaled	self-assessed mental exhaustion or burnout	might reduce the perceived level of mental fatigue or burnout
Taavoni et al. [70]	lavender, geranium, rose, and rosemary	massage	the menopause rating scale	reduced psychological symptoms
Seol et al. [71]	*Salvia sclarea* and lavender	inhaled	a questionnaire	lowered stress during urodynamic examinations and induced relaxation in female urinary incontinence patients undergoing urodynamic assessments
Fu et al. [74]	lavender	inhaled	mini mental state examination and the CMAI short form	reduced disruptive behavior
Apay et al. [76]	lavender	massage	visual analog scale	effect of aromatherapy massage on pain was higher than that of placebo massage

### 2.4. Mechanism Studies from Basic Research

Lavender was one of the essential oils that was completely researched in terms of its safety and mechanisms in humans (Table 4). From the imaging study, it was found that Silexan intake, a patented product of lavender oil, showed a reduction in serotonin-1A receptor binding in several brain areas. *Acori Tatarinowii* Rhizoma showed a synergistic effect with the nerve growth factor in pheochromocytoma PC12 cells potentiating neurite outgrowth in an essential oil co-treatment group. Menthol could increase the circadian eating behavior in animal research. The treatment of alpha (α)- and beta (β)-pinene could reduce the nitrite level in the hippocampus and lower dopamine and norepinephrine levels in the striatum, resulting in seizure intensity reduction. *Ocimum gratissimum* essential oil showed anesthetic properties and reduced stress in Nile tilapia during transport. Coriander oil and linalool showed a sedative effect, reducing stress-related behaviors in chicks in a manner similar to the effects of diazepam. Lavender, peppermint, rosemary, grapefruit, bergamot, and yuzu could modulate autonomic nervous system function, resulting in changes in cardiovascular parameters and cortisol release. Citronellol showed a nociceptive effect of orofacial pain via the retrosplenial cortex and periaqueductal gray activations. Bergamot oil also reduced the central sensitization-phase-related pain and agitation behaviors in a mouse model. Peppermint essential oil reduced mental fatigue and prolonged cognitive tasks with acetylcholinesterase inhibitory and gamma-aminobutyric acid A receptor stimulating properties. Geraniol oil also improved learning and memory impairment related to aging.

**Table 4:** Studies related mechanisms of essential oils.

Author	Essential Oils	Application Methods	Measures	Outcomes
Scuteri et.al. [8]	bergamot	inhaled	licking/biting behavior	analgesic properties
Du et al. [13]	lemon and grapeseed	inhaled	cognitive function tests	shortened reaction time response, and more impulsive decision-making.
Dehghan et al. [14]	lavender, rosemary, and orange	inhaled	retrospective and prospective memory scale	only lavender or rosemary could reduce some memory problems in hemodialysis patients by reduction of retrospective memory problems
Chen et al. [15]	bergamot	inhaled	a questionnaire including the Edinburgh postnatal depression scale and postpartum sleep quality scale (PSQS)	alleviated depressive mood in postpartum
Atef et al. [16]	geraniol	oral	Morris water maze test	shortened escape latency and increased platform crossing
Ferreira et al. [26]	*Ocimum gratissimum*	water medication (immersion)	the time of anesthesia induction and recovery during anesthesia of *Oreochromis niloticus* exposed to essential oil of *Ocimum gratissimum*	reduced the stress of transport and improved the oxidative status of *Oreochromis niloticus* by stable plasma glucose and change antioxidant defense system by increasing hepatic and kidney ROS
Schneider [28]	peppermint, rosemary, and grapefruit	inhaled	the hot immersion test paradigm and physiological parameters	resisted a stressful thermal stimulus and increased HRV
Hacke et al. [29]	lemongrass, pure citral and geraniol	water medication (immersion)	the light–dark test	anxiolytic effect
Kawai et al. [30]	grapefruit	inhaled	muscle sympathetic nerve activity (MSNA), blood pressure (BP), heart rate (HR), and cortisol concentration	changed in BP and MSNA as well as decreased stress hormone cortisol
Felipe et al. [36]	alpha-pinene and beta-pinene	oral	determination of dopamine and norepinephrine content, thiobarbituric acid reactive substances, and nitrite concentration	reduced nitrite level and norepinephrine and dopamine (NE-DA) content during pentylenetetrazole-induced seizure
Qadeer et al. [41]	lavender	oral	open field test (OFT), light/dark transition box activity, forced swim test (FST) and corticosterone, lipid peroxidation, and endogenous antioxidant enzymes activities	stress induced behavior and biochemical alteration in rats
Kennedy et al. [44]	spearmint and peppermint	oral	neurotransmitter receptor binding, acetylcholinesterase (AChE) inhibition, mood scales, and a standardised cognitively demanding tasks	peppermint with menthol showed in vitro cholinergic inhibitory, calcium regulatory, GABA, nicotinic binding effect; modulated performance on demanding cognitive task; attenuated the increase in mental fatigue associated with extended cognitive task
Lam et al. [49]	*Acori Tatarinowii* Rhizoma, *Acori Graminei* Rhizoma, and *Acori Calami* Rhizoma	exposure in cell culture media	transcriptional activation of neurofilament promoters and the neurite outgrowth	potentiated nerve growth factor (NGF)-induced neuronal differentiation in PC12 and neurite outgrowth-neurofilament expression
Chen et al. [53]	lavender	massage	salivary cortisol and Immune function measures	decreased stress and enhanced immune function
Gaston et al. [55]	*Coriandrum sativum*	intracerebroventricular injection	OFT test	sedative effect
Watanabe et al. [58]	bergamot	inhaled	salivary cortisol level	lowered salivary cortisol compared to rest
Hasanein and Riahi [60]	lemon balm	injection	nociceptive test	treatment of painful diabetic neuropathy
Matsumoto et al. [64]	yuzu	inhaled	the profile of mood states (POMS) questionnaire and salivary chromogranin A	alleviated negative emotional stress-suppression of sympathetic nervous system activity
Han et al. [73]	*Acorus tatarinowii* Schott	injection/intraperitoneal	OFT, FST, and tail suspension test (TST)	essential oils and asarones from the rhizomes of *Acorus tatarinowii* could be considered as a new therapeutic agent for curing depression
Brito et al. [75]	citronellol	paw injection	nociceptive test	attenuated orofacial pain

## 3. Discussion

In this scoping review, we updated the effects of essential oils on the nervous system for this decade in terms of physiological and pathophysiological conditions. The number of studies on essential oils is increasing year by year, and more mechanisms are being revealed. The PubMed search strategy showed more specific research compared with Scopus and Google Scholar. However, a number of studies were found in the Scopus and Google Scholar databases. In this review, we intended to provide a comprehensive view of this issue. Therefore, we included both animal and human research to completely explain the beneficial properties and related mechanisms. We did not perform risk of bias in this systematic scoping review. Most of the excluded studies were not related to nervous system function and were written in other languages. The population covered in this review is broad, as reported in Table 1, to show the previous use of essential oils in many possible models. We analyzed the included studies and categorized the physiological and pathophysiological modifications.

It is not only the olfactory system that plays a role in this intervention; the direct effect of odorant compounds also takes part in the central nervous system [77]. The intervention mechanisms might be split into two forms: action via the olfactory system and action via the oil’s own chemical properties. Actions in both physiology and pathophysiology ranges through the olfactory system connect with the hypothalamus, limbic system, and prefrontal cortex, exerting body responses [61]. The suprachiasmatic nucleus (SCN) plays an important part in our sense of smell [78]. In the hypothalamus–hypophysis–adrenal axis, essential oils and emotional signals from the prefrontal cortex, amygdala, and hippocampus could reduce corticotropin-releasing hormone (CRH), which then reduced the adrenocorticotropic hormone (ACTH). The reduction in ACTH leads to a lower release of stress hormone as cortisol in serum [30,53,58]. Autonomic nervous system activity from odorants is also involved with SCN, which in turn reduces sympathetic activity and increases parasympathetic activity. Endogenous opioids are an important factor in mental issues [79]. These activities were observed using cardiovascular parameters and the responses of other organs such as the pupils, skin, and gastrointestinal system, or based on cerebral activity [80]. In the physiological range, most of the essential oils used in research cause body responses in a parasympathetic fashion. Interestingly, menthol can cause a cold perception triggered by transient receptor potential channel (TRP) stimulation. This psychological perception of TRP could increase the sympathetic response and food intake [81]. Another psychological aspect is distraction, and aromatherapy also successfully distracted patients or participants from anxiety, stress, and pain [31]. Regarding the chemical properties of essential oils, many receptors of neurotransmitters are involved, such as gamma-aminobutyric acid (GABA) A receptor, n-methyl-D-aspartate (NMDA) receptor, serotonin (5-HT) 1A receptor, and a voltage-dependent calcium channel. For arousal, GABA and NMDA receptors take part in the function causing sedative or stimulating effects. Linalool increased the chloride current from GABA receptor stimulation, which then caused sedation. For depressive symptoms, 5-HT is the ideal neurotransmitter system used in antidepressants. Silexan showed a reduction in 5-HT1A receptor binding after oral route administration for 8 weeks [68]. This action might be the mechanism of anxiolytic and antidepressant activities. Essential oils still have a direct effect on the nociceptor in case of analgesic effect [82].

Regarding the null results in children with burns, breast reconstruction patients, and open-heart surgery patients, there are several explanations in these cases [18,39,50]. Very young children showed a low level of stress compared with adults or older children because they could comfort themselves by being close to their parents. Intense distress can happen as a result of cancer diagnoses and surgery causing concerns about a lower household income, reduced social support, and higher tumor stage. There is a strong level of fear associated with acute procedures involving invasive interventions such as open-heart surgery. This scoping review showed broad, complex health outcomes from basic mechanisms to pathophysiology through a key strategic search. There is a limitation related to the outcome measures related to explicit measures based on rating methodology. Objective measures were used for most of the physiological parameters. To compare efficacy among EOs, the dose of EOs, duration of application, risk of bias, and measures need to be comparable. The objective or implicit measures will provide a benefit when comparing efficacy. Altogether, essential oils showed beneficial effects on the nervous system in both physiological and pathological ranges. Concern about pathologic mechanisms and actions of essential oils in terms of types, as well as the administration route, will benefit wellbeing and quality of life.

## 4. Materials and Methods

### 4.1. Question

Preferred reporting items for systematic reviews and meta-analyses extension for scoping reviews were used as guidelines during the preparation of this scoping review. The scoping review intended to update the comprehensive evidence to answer the following question: “What are the effects of essential oils on the nervous system?”

### 4.2. Search Strategy

To completely answer the question, we set up a strategic search concerning sensitivity and specificity to identify literature related to the nervous system. Three electronic databases—PubMed, Scopus, and Google Scholar—were used in this study, which was conducted by two independent reviewers (AS and PK) on 5 October 2022. A search strategy for PubMed was created using a combination of MeSH terms and Boolean operators “AND”, “OR”: ((Oils, Volatile[MeSH Terms]) OR (essential oil*[Title/Abstract])) AND (((((((((Nervous System[MeSH Terms]) OR (nervous system*[Title/Abstract])) OR (cognition[MeSH Terms])) OR (consciousnesses[MeSH Terms])) OR (arousal[MeSH Terms])) OR (arousal*[Title/Abstract])) OR (behavior[MeSH Terms])) OR (behavior*[Title/Abstract])) OR (executive function[MeSH Terms])) filters: from 2012 to 2022. For other databases, the searching strategy was partially modified. Articles published from January 2012 onward were included in the acquisition process. Included studies were further screened, and a manual search was performed to discover any missing data. Endnote 20 (PDFNet SDK © PDFTron™ Systems Inc., 2001–2020, and distributed by Clarivate Analytics (US) LLC under license) was used as an imported and managing software.

### 4.3. Study Selection

Duplicated studies were filtered using Endnote. Two reviewers (AS and PK) independently screened the titles and abstracts of all studies and subjected them to the inclusion and exclusion criteria. For the inclusion criteria, original articles written in English and describing animal and human studies were selected for every age group and health condition. For the exclusion criteria, articles published in other languages and including no outcome of the nervous system were excluded. Then, the full texts were downloaded and evaluated. Any disagreements during the selection processes were resolved via discussion and consensus between the two writing reviewers.

### 4.4. Data Charting Process

The data extraction process was performed by two writing reviewers (AS and PK). A standardized data extraction form including the author, year, study design, type of essential oils, route of administration, population, outcome measure methodology, and outcome was extracted using Microsoft Excel software. Any disagreements during the selection processes were resolved via discussion and consensus between the two writing reviewers. Tables listing the essential oils and outcomes were created to conclude the selected studies.

## 5. Conclusions

Essential oils showed beneficial effects on the nervous system in both animal and human research via the olfactory system and its chemical properties, impacting physiology and pathophysiology.

## Data Availability

Not applicable.
